# ﻿A new species of the genus *Liotyphlops* Peters, 1881 (Serpentes, Anomalepididae) from Colombia and the synonymization of *Liotyphlopsbeui* (Amaral, 1924) with *Liotyphlopsternetzii* (Boulenger, 1896)

**DOI:** 10.3897/zookeys.1146.94607

**Published:** 2023-02-07

**Authors:** Fidélis Júnio Marra Santos

**Affiliations:** 1 Pontifícia Universidade Católica do Rio Grande do Sul (PUCRS), Laboratory of Vertebrate Systematics. Av. Ipiranga, 6681 Partenon; 90619-900, Porto Alegre, Rio Grande do Sul, Brazil Pontifícia Universidade Católica do Rio Grande do Sul (PUCRS) Porto Alegre Brazil

**Keywords:** Biodiversity, *Liotyphlopspalauophis* sp. nov., neotropics, reptiles, Scolecophidia, taxonomy

## Abstract

A new species of *Liotyphlops* Peters, 1881, *Liotyphlopspalauophis***sp. nov.**, is described from the neighborhoods of Bogota, Colombia from a previous syntype of *L.anops*, and a lectotype is designated for the latter species. The new species is readily distinguished from congeners by having the frontal scale divided (vs single), and a central foramen in the parabasisphenoid (vs foramen absent). High-resolution x-ray computed tomography (HRXCT) was used to study and present data on the skull of the holotype of the new species, the lectotype of *L.anops*, and the holotype of *L.ternetzii*. Additionally, extensive study of skull characters and external morphology failed to find diagnostic characters to differentiate *L.beui* and *L.ternetzii*, and the former is here considered a junior synonym of *L.ternetzii*, which is also redescribed.

## ﻿Introduction

The genus *Liotyphlops* Peters, 1881 is a group of small, cryptozoic blindsnakes, distributed in the Neotropics, from Costa Rica to Argentina. *Liotyphlops* is currently composed of 13 species (Santos and Reis 2018; Boundy 2021; Linares-Vargas et al. 2021): *Liotyphlopsalbirostris* (Peters, 1858); *L.anops* (Cope, 1899); *L.argaleus* Dixon & Kofron, 1984; *L.beui* (Amaral, 1924); *L.bondensis* (Griffin, 1916); *L.caissara* Centeno, Sawaya & Germano, 2010; *L.haadi* Silva-Haad, Franco & Maldonado, 2008; *L.schubarti* Vanzolini, 1948; *L.sousai* Santos & Reis, 2018, *L.taylori* Santos & Reis, 2018, *L.ternetzii* (Boulenger, 1896); *L.trefauti* Freire, Caramaschi & Argôlo, 2007, and *L.wilderi* (Garman, 1883). Brazil has the greatest diversity of *Liotyphlops* snakes, with eight valid species. In recent years, the description of new species of Anomalepididae have been restricted to the genus *Liotyphlops* (Freire et al. 2007; Haad et al. 2008; Centeno et al. 2010; Santos and Reis 2018) and revalidations of supposed synonyms of *L.albirostris* (Linares-Vargas et al. 2021). In the present study, an additional new species of *Liotyphlops* is described from Colombia.

*Helminthophisanops* was described by Cope (1899) based on two specimens; he wrote: “The collection which furnishes the basis of the investigation presented in the following pages was made in Colombia, near Bogota, for the World’s Exposition of Chicago, where it was exhibited in the department of New Granada. The number of species is fifty-four, of which nine are new to science. I have not been able to ascertain the exact localities at which the specimens were obtained, but most of them, it is believed, were found in the neighborhood of Bogota” (Cope 1899: 3). Subsequently, Dunn (1944) transferred *H.anops* to *Liotyphlops*, also in Anomalepididae. Cope (1899: 10–11) also wrote: “This species has a tendency to subdivision of scales. In one of the two specimens the frontal is divided into two regular scales, and in another the lower extremity of the first labial is cut off on one side”. The holotype of the new species described here (AMNH R-9550) is one of the two syntypes of *H.anops* and distinct from the other syntype (AMNH R-17540) in possessing, among other diagnostic characters, the frontal scale divided (vs single), and a central foramen in the parabasisphenoid (vs foramen absent). The other syntype (AMNH R-17540) is consistent with the species currently identified in Colombia as *L.anops*.

Taxonomic changes over the past century have also included two other species of *Liotyphlops*: *L.beui* and *L.ternetzii*. The original description of *L.ternetzii*, by Boulenger (1896, as *Helminthophisternetzii*) was based on a single specimen from “Paraguay” (holotype BMNH 1946.1.11.77). Later, Smith and Grant (1958) recognized *Liotyphlops* as a genus distinct from *Helminthophis*, highlighting as a diagnostic character the separation of prefrontal scales in *Liotyphlops*, while in *Helmintophis* the prefrontal scales are widely in contact. They transferred Boulenger’s species to *Liotyphlops*. Boulenger (1896: 584) characterized this species as: “rostral two fifths the width of the head, extending nearly to the level of the eyes, forming a broad, straight suture with the frontal, which is about twice as broad as long; eye scarcely distinguishable through the ocular; two superposed preoculars and a subocular; four upper labials, first largest, second and third in contact with the lower preocular, third and fourth in contact with the subocular. Diameter of body 52 times in total length; tail nearly twice as long as broad, ending in a spine. 22 scales round the body. Olive above and beneath; head and anal region yellowish. Total length 335 mm.”

*Liotyphlopsbeui* was originally described by Amaral (1924), as *Helminthophisbeui* from Butantan, São Paulo, Brazil (holotype IB 1806 and paratypes IB 281, IB 282, IB 652, and IB 1041). Amaral (1924: 29) characterized his new species as: “snout acutely rounded; rostral about two fifths the width of the head, not extending posteriorly to the vertical plane of the eyes, rounded posteriorly and forming a narrow suture with the frontal; frontal only about three times as wide as long; one subocular; two preoculars; eye under the suture between the ocular and lower preocular; four upper labials, 1^st^ largest, 2^nd^ and 3^rd^ in contact with the subocular, which separates them from the lower preocular; prefrontal separated from the 2^nd^ labial by the lower preocular, nasal and subocular. Tail more than twice as long as broad, ending in a spine. 22 scale rows around the body. Dark brown to blackish brown; head, as well as anal region and surroundings, light yellow; terminal spine yellowish. Total length, 290 mm; tail, 10 mm.” Only five years after the original description, *H.beui* was placed in the synonymy of *H.ternetzii* by Amaral (1929) himself, but 55 years later Dixon and Kofron (1984) believed the species was valid and removed it from the synonymy of *L.ternetzii* based on the possession of 20 scale rows around the posterior body (22 in *L.ternetzii*) and fewer dorsal scales, 384–455 (vs 463–510 in *L.ternetzii*). Despite some authors maintaining *L.beui* as synonym of *L.ternetzii* (e.g., Peters et al. 1986), most subsequent authors have followed Dixon and Kofron (1984) and treated *L.beui* as a valid species (McDiarmid et al. 1999; Freire et al. 2007; Haad et al. 2008; Centeno et al. 2010; Wallach et al. 2014; Santos and Reis 2018; Boundy 2021; Linares-Vargas et al. 2021).

Here it is important to highlight the research of Dixon and Kofron (1984). They observed that most of the characters utilized for described forms are variable within populations, and occasionally the squamation is different on each side of the head in an individual. Also, according to Dixon and Kofron (1984), the nasal scale is divided and is variously called upper and lower nasals, preseminasals and postseminasals, anterior nasals and postnasals, or just nasals; additionally, the lateral and dorsomedian head scales are variously called subocular(s), preocular(s), ocular, supraocular(s), frontal, and postfrontal(s). They explained that much depends upon one’s concept of the position of the scales as to whether there are two suboculars and one preocular, or two preocular and one subocular, or two supraoculars and one preocular, or two preoculars and one supraocular, etc. Accordingly to Dixon and Kofron (1984) the presence or absence of the division and/or fusion of scales on one side of the head and not on the other has been largely ignored by most describers of *Liotyphlops* species, which has, therefore, resulted in poor species concepts; the only scales that appear to be consistently defined in all writings are the rostral, prefrontal, and frontal scales.

In this paper, the validity of *L.beui* is revisited and *L.ternetzii* is redescribed. A new species of *Liotyphlops* is also described from the neighborhoods of Bogota, Colombia, a lectotype is designated for *L.anops*, and that lectotype is also redescribed. High-resolution x-ray computed tomography (HRXCT) was used to present data on the skull of the holotype of *L.ternetzii* and the holotype of the new species.

## ﻿Materials and methods

I adopted the definition of the Unified Species Concept (Queiroz 2007), in which species are equated with independently evolving metapopulation lineages. In the absence of autapomorphy for species, consistent morphological difference among separate populations is used as a proxy for lineage independence. The study of external morphology was conducted under a stereomicroscope. The terminology used for the head squamation and scale counts follows Dixon and Kofron (1984) and Santos and Reis (2018). Measurements were taken with digital calipers and are presented as percent of total length (TL), except for subunits of the head, which are presented as percent of head length (HL). The results of morphometric analyzes are presented in the description. Specimens were not sexed and only adult specimens were examined (see Appendix 1). The photographs were obtained using a digital Nikon D5100 camera. For drawing preparation, a Wacom Intuos Draw CTL490DW digital tablet was used with the desktop digital stereomicroscope COSMOS LCD.

For the comparisons of *Liotyphlopsternetzii* and *L.beui*, the holotype of the former and paratypes of the latter were used. In addition, 50 specimens of each of these two species were measured and counted for the comparisons.

The head of the holotype of *L.ternetzii* and paratype of *L.beui* were studied by high-resolution x-ray computed tomography (HRXCT) at the high-resolution x-ray CT facility of the University of Texas at Austin using an Xradia microCT Scanner, and the holotype of the new species of *Liotyphlops* was studied by HRXCT at the high-resolution x-ray CT facility at Pontifícia Universidade Católica do Rio Grande do Sul using a Skyscan 1173 microfocus x-ray CT. The datasets were rendered in three dimensions using CTvox v. 3.2 (Bruker microCT, Inc., Billerica, MA) for Windows.

The terminology used for bones follows Rieppel et al. (2009), Santos and Reis (2018), and Santos and Reis (2019). The locality of the specimens was plotted using Google Earth Pro v. 7.3.2.5495, and the map was built with ArcMap (ArcGis) v. 10.4.1 for desktop using the WGS1984 geodetic datum. Geographical coordinates for historical specimens with imprecise locality records were approximated using the best evidence available and plotted with Google Earth. Only specimens actually examined were used in the map. Institutional abbreviations of specimens examined follow Sabaj (2020), with the addition of CEPB (Centro de Estudos e Pesquisas Biológicas da Pontifícia Universidade Católica de Goiás, Goiânia, Brazil).

## ﻿Results

### ﻿Taxonomic account

#### ﻿ Liotyphlops palauophis sp. nov.

Taxon classificationAnimaliaSquamataAnomalepididae

A7FC4D63-F781-5CC1-BA31-DD6FA74986DA

https://zoobank.org/0CD30C35-2263-491C-BB62-2494019740C8

**Figure 1.**
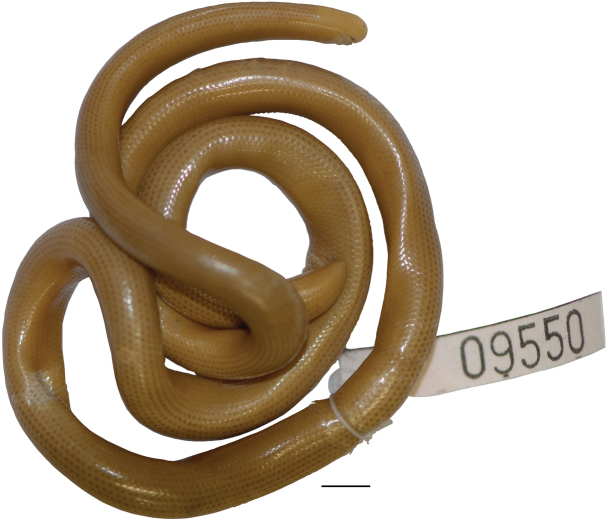
Holotype of *Liotyphlopspalauophis* sp. nov., AMNH R-9550, 361.2 mm TL, Colombia, neighborhood of Bogota. Scale bar: 5 mm.

**Figure 2.**
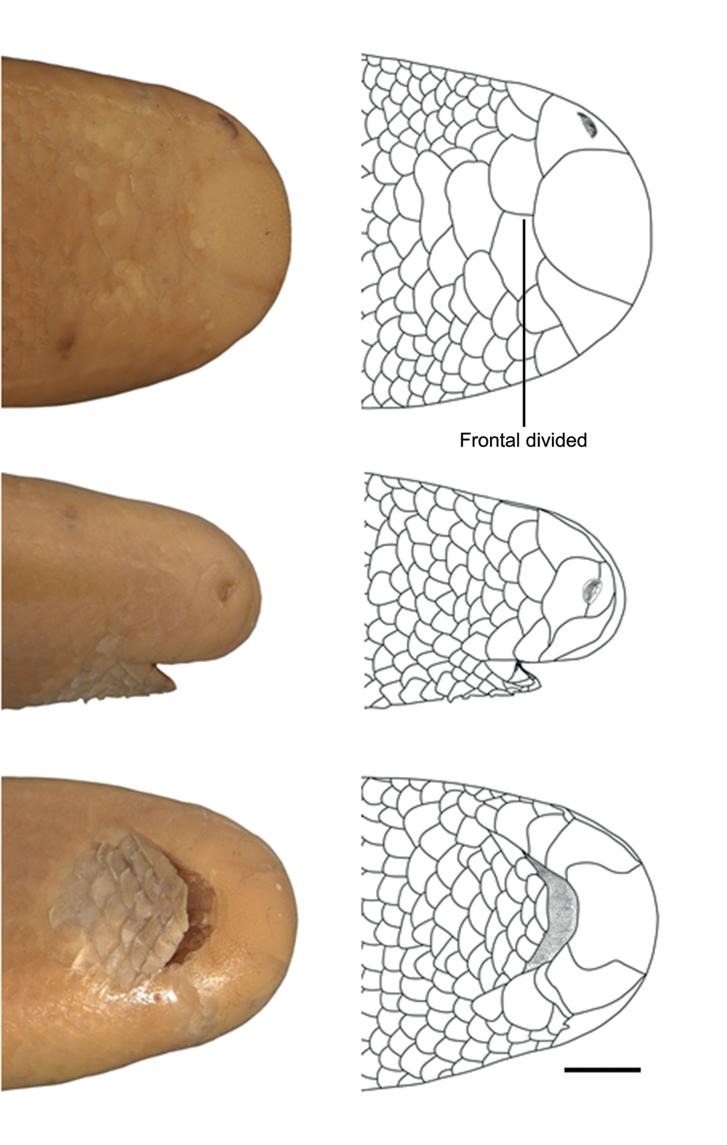
Dorsal (top), lateral (center), and ventral (bottom) views of the head of *Liotyphlopspalauophis* sp. nov., AMNH R-9550, holotype. Scale bar: 1 mm.

**Figure 3.**
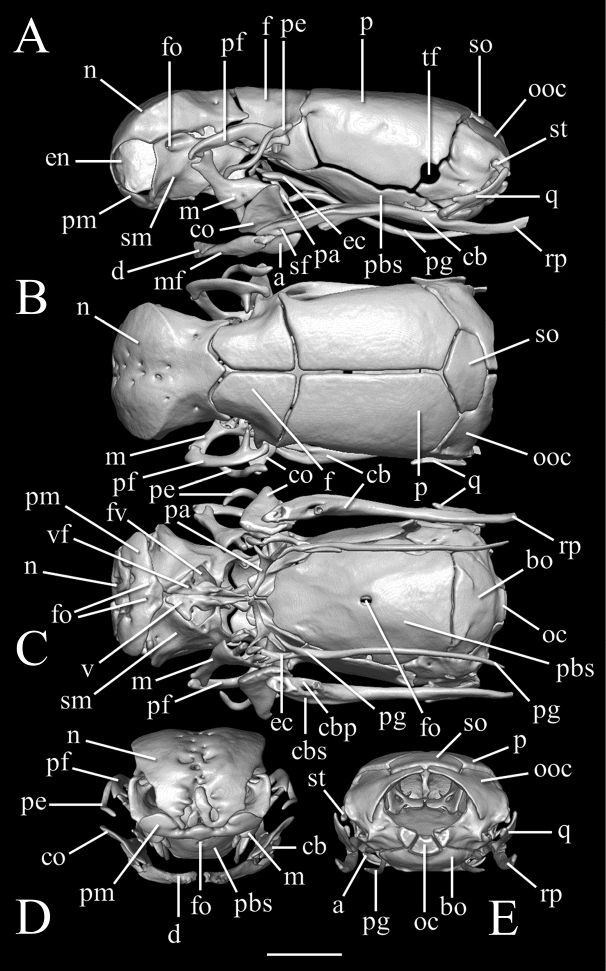
Three-dimensional reconstruction of the skull of *Liotyphlopspalauophis* sp. nov., AMNH R-9550, holotype, based on HRXCT data. **A** lateral view **B** dorsal view **C** ventral view with lower jaw partially digitally removed **D** anterior view **E** posterior view. Scale bar: 1 mm. Anatomical abbreviations: **a**: angular; **bo**: basioccipital; **cb**: compound bone; **co**: coronoid; **d**: dentary; **ec**: ectopterygoid; **en**: external naris; **f**: frontal; **fo**: foramen; **fv**: fenestra vomeronasalis; **m**: maxilla; **mf**: mental foramen; **n**: nasal; **oc**: occipital condyle; **ooc**: otico–occipital (fused prootic + opisthotic + exoccipital); **p**: parietal; **pa**: palatine; **pbs**: parabasisphenoid; **pe**: postorbital element; **pf**: prefrontal; **pg**: pterygoid; **pm**: premaxilla; **q**: quadrate; **rp**: retroarticular process; **sf**: surangular foramen; **sm**: septomaxilla; **st**: supratemporal; **so**: supraoccipital; **tf**: trigeminal foramen; **v**: vomer; **vf**: vomerine foramen.

**Figure 4.**
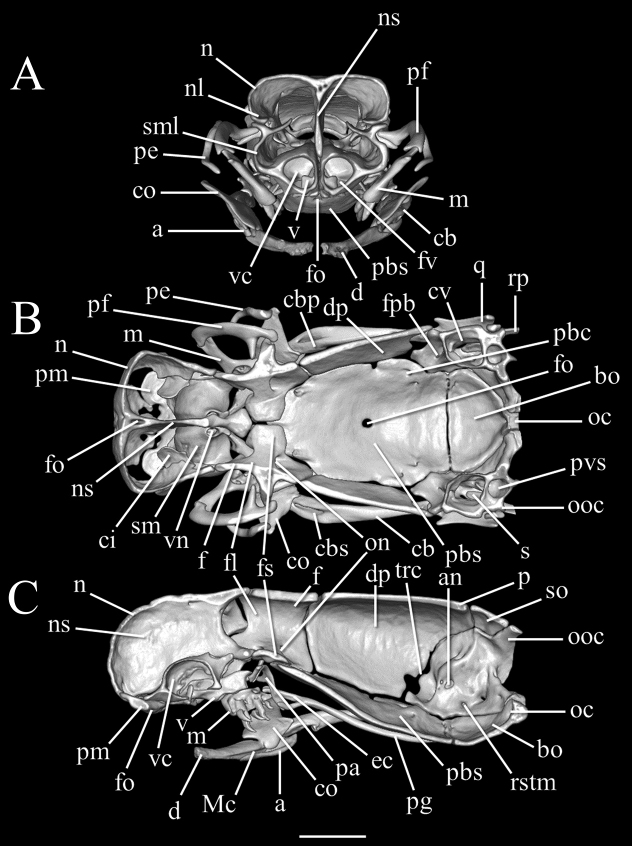
Three-dimensional reconstruction of the skull of *Liotyphlopspalauophis* sp. nov., AMNH R-9550, holotype, based on HRXCT data. **A** transversal view **B** frontal view **C** sagittal view. Scale bar: 1 mm. Anatomical abbreviations: **a**: angular; **an**: acoustic nerve foramen; **bo**: basioccipital; **cb**: compound bone; **cbp**: compound bone prearticular component; **cbs**: compound bone surangular component; **ci**: conchal invagination; **co**: coronoid; **cv**: cavum vestibuli; **d**: dentary; **dp**: descensus parietalis; **ec**: ectopterygoid; **en**: external naris; **f**: frontal; **ﬂ**: frontal laterally descending ﬂange; **fo**: foramen; **fpb**: facial nerve palatine branch foramen; **fs**: frontal subolfactory process; **fv**: fenestra vomeronasalis; **m**: maxilla; **Mc**: Meckel’s canal; **mf**: mental foramen; **n**: nasal; **nl**: nasal lateral ﬂange; **ns**: medial nasal septum; **oc**: occipital condyle; **on**: optic nerve foramen; **ooc**: otico–occipital (fused prootic + opisthotic + exoccipital); **p**: parietal; **pa**: palatine; **pbc**: parabasal (Vidian) canal; **pbs**: parabasisphenoid; **pe**: postorbital element; **pf**: prefrontal; **pg**: pterygoid; **pm**: premaxilla; **pvs**: posterior vertical semicircular canal; **q**: quadrate; **rp**: retroarticular process; **rstm**: recessus scalae tympani medial aperture; **s**: stapes; **sf**: surangular foramen; **sm**: septomaxilla; **sml**: septomaxilla lateral ﬂange; **st**: supratemporal; **so**: supraoccipital; **tf**: trigeminal foramen; **trc**: trigeminofacialis chamber; **v**: vomer; **vc**: vomeronasal cupola; **vf**: vomerine foramen; **vn**: vomeronasal nerve passage.

**Figure 5.**
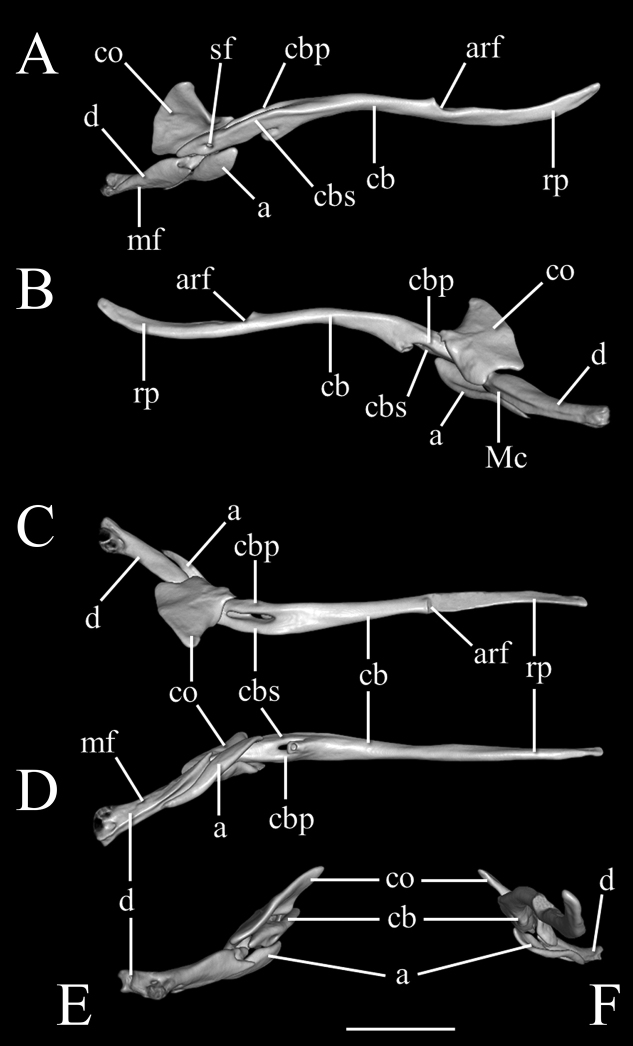
Three-dimensional reconstruction of the lower jaw of *Liotyphlopspalauophis* sp. nov., AMNH R-9550, holotype, based on HRXCT data. **A** lateral view **B** medial view **C** dorsal view **D** ventral view **E** anterior view **F** posterior view. Scale bar: 1 mm. Anatomical abbreviations: **a**: angular; **arf**: articular fossa; **cb**: compound bone; **cbp**: compound bone prearticular component; **cbs**: compound bone surangular component; **co**: coronoid; **d**: dentary; **Mc**: Meckel’s canal; **mf**: mental foramen; **rp**: retroarticular process; **sf**: surangular foramen.

**Figure 6.**
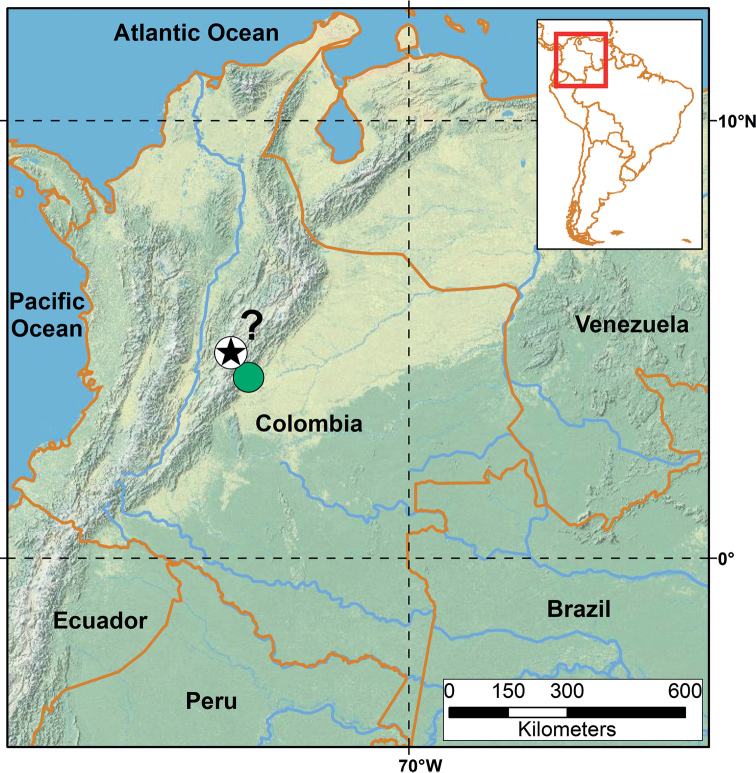
Location of the holotype of *Liotyphlopspalauophis* sp. nov. (black star), lectotype of *Liotyphlopsanops* (white dot), and specimens of *L.anops* examined in this study (green dot). ? = lack of detailed information about the type locality of *L.palauophis* sp. nov. and *L.anops*. This locality is based on information provided by Cope (1899).

Figs 1 , 2 , 3 , 4 , 5 , 6 , Table 1

Helminthophis anops Cope, 1899 (in part). Syntype of H.anops.

##### Type material.

***Holotype*.** AMNH R-9550, 361 mm TL, Colombia, neighborhood of Bogota, 1899.

##### Diagnosis.

*Liotyphlopspalauophis* sp. nov. is distinguished from all other *Liotyphlops* by having the frontal scale divided (vs single) and a central foramen in the parabasisphenoid (vs foramen absent). It is further distinguished from *L.albirostris*, *L.argaleus*, *L.bondensis*, *L.caissara*, *L.haadi*, *L.trefauti*, and *L.wilderi* in having two scales (vs one scale) contacting the posterior edge of the nasal between the second supralabial and prefrontal. It is further distinguished from *L.beui*, *L.schubarti*, *L.taylori*, and *L.ternetzii* by having four (vs three) scales contacting the posterior edge of the prefrontal. It is distinguished from *L.anops* by having 28/26/26 scales around the body and 19 subcaudal scales (vs 26/24/24 scales around the body and 12–14 subcaudal scales), and from *L.sousai* in having 573 dorsal scales and 561 ventral scales (vs 439 dorsal scales and 427 ventral scales).

##### Description.

Meristic data in Table 1. Total length 361.2 mm, head length 5.3 mm (1.5% TL), snout–vent length 353 mm (97.7% TL), tail length 8.2 mm (2.3% TL), head width 3.8 mm (71.7% HL), and head height 3.1 mm (58.5% HL). Body covered with cycloid scales. Rostral scale large, longer than wide, contacting nasals anterolaterally, prefrontals laterally, and divided frontal posteriorly. Pair of triangular prefrontals bordered anterolaterally by rostral, ventrally by large divided nasal, and dorsoposteriorly by frontal. Posterior edge of prefrontals passing posterior edge of rostral. Frontal scale divided. Nasal scale divided and bordered anteriorly by rostral, dorsally by prefrontal, ventrally by first and second supralabials, and posteriorly by two scales that lie between prefrontal and second supralabial. Eye spot not visible. Four scales contacting posterior edge of prefrontal (three cycloid scales + frontal). Two scales contacting posterior edge of nasal between second supralabial and prefrontal. Six scales in first vertical row of dorsal scales. Mental triangular, not divided, wider than long, contacting first infralabials. Supralabials four, infralabials three. Scales around body 28/26/26. Dorsal scales 573, vental scales 561, and subcaudal scales 19.

**Table 1.** Meristic characters of species of *Liotyphlops* from the specimens examined in this study, presented as ranges with minimum, maximum, and mode in parentheses. **SPEP** = number of scales contacting posterior edge of prefrontal; **SPEN** = number of scales contacting posterior edge of nasal between second supralabial and prefrontal; **SFVRD** = number of scales in the first vertical row of dorsals; **SL** = number of supralabial scales; **IL** = number of infralabial scales; **ASR** = number of anterior scale rows around body; **MSR** = number of scale rows around the midbody; **PSR** = number of posterior scale rows around body; **DSR** = number of dorsal scale rows; **VSR** = number of ventral scales rows; **SC** = number of subcaudal scales. **n** = number of specimens examined in this study. **^a^** = number of specimens examined by Santos and Reis (2018). **^b^** = number of specimens examined by Centeno et al. (2010). **^c^** = number of specimens examined by Freire et al. (2007). **^d^** = number of specimens examined by Linares-Vargas et al. (2021).

Species/Count	n	SPEP	SPEN	SFVRD	SL	IL	ASR	MSR	PSR	DSR	VSR	SC
*L.albirostris* **^a^**	6	3–3(3)	1–1(1)	5–5(5)	4–4(4)	3–3(3)	24–26(26)	22–22(22)	22–22(22)	432–478	417–453	12–17(12)
*L.anops* **^a^**	3	4–4(4)	2–2(2)	5–6(5)	4–4(4)	3–3(3)	26–26(26)	24–24(24)	24–24(24)	562–597	531–572	12–14
*L.argaleus* **^a^**	1	4	1	4	4	3	25	23	22	497	472	16
*L.beui* **^a^**	50	3–3(3)	2–2(2)	5–6(5)	4–4(4)	3–3(3)	22–26(22)	20–22(22)	20–22(20)	366–532(453)	348–511(364)	11–22(12)
*L.bondensis* **^d^**	17	3	1	4	4	3	24	22	22	363–449	347–434	11–17
*L.caissara* **^b^**	1	3	1	4	3	3	22	20	20	326	308	10
*L.haadi* **^a^**	2	3–3(3)	1–1(1)	4–4(4)	4–4(4)	3–3(3)	20–20(20)	19–20	18–20	333–384	309–348	11–12
*L.palauophis*	1	4	2	6	4	3	28	26	26	573	561	19
*L.schubarti* **^a^**	5	3–3(3)	2–2(2)	5–5(5)	4–4(4)	3–3(3)	22–24(22)	20–22(20)	20–20(20)	417–463	398–451	11–14(13)
*L.sousai* **^a^**	1	4	2	6	4	3	24	22	20	439	427	13
*L.taylori* **^a^**	1	3	2	5	4	2	22	20	20	455	441	14
*L.ternetzii* **^a^**	50	3–3(3)	2–2(2)	5–6(5)	4–4(4)	3–3(3)	22–26(22)	20–23(20)	20–22(20)	353–539(417)	341–514(381)	11–22(15)
*L.trefauti* **^c^**	2	4–4(4)	1–1(1)	5–5(5)	4–4(4)	4–4(4)	22–22(22)	22–22(22)	22–22(22)	520–543	499–531	8(8)
*L.wilderi* **^a^**	3	3–3(3)	1–1(1)	4–4(4)	4–4(4)	3–3(3)	22–24(22)	22–22(22)	20–21(20)	385–402	371–383	12–19(12)

##### Description of skull.

High-resolution x-ray computed tomography of skull bones in Figs 3–5. Main body of premaxilla on ventral surface of snout. Maxilla–premaxilla contact widely separated. Lateral maxillary foramina absent. Maxilla alveolar row oriented transversely. Nasal fused. Nasal–frontal boundary convex posteriorly in a shallow W-shaped suture. Prefrontal separated from nasal. Prefrontal moveably articulated to frontal. Postorbital element present. Posterior orbital margin incomplete. Frontals gradually tapering anteriorly. Frontal paired. Frontal–parietal contact (dorsal aspect) mostly straight and transverse, median notch in frontals slight at most. Parietal paired. Posterior border of parietal without median projection over supraoccipital. Supratemporal present. Posteromedial flange of septomaxilla short, not contacting frontal. Septomaxilla with lateral flange contributing to posterior border of external naris. Fenestra for duct of Jacobson’s organ posteroventrally positioned. Palatine not in contact with vomer, maxilla, or pterygoid. Central foramen present in parabasisphenoid. Ectopterygoid present. Supraoccipital present and single not participating in internal sidewall of neurocranium. External surface (dorsoposterior) of supraoccipital without transverse ridge. Supraoccipital–prootic contact narrow, less than half supraoccipital–parietal contact. Splenial not present as discrete element. Coronoid and angular separated by prearticular portion of compound bone. Retroarticular process long, longer than articular facet. Teeth present in maxilla, but lacking in dentary, premaxilla, palatine, and pterygoid.

##### Coloration in alcohol.

Dorsal and ventral body pale cream with brown pigmentation points along dorsal region of body.

##### Distribution.

Known only from the type locality in the neighborhood of Bogota, Colombia (Fig. 6), according to the information provided by Cope (1899).

##### Etymology.

The species name is in honor of Alfredo Palau Peña (June 10, 1969–August 8, 2020), a Brazilian herpetologist and my friend, who was killed by the COVID-19 virus. A combination of his name *Palau* and the Greek *ophis*, meaning snake.

#### ﻿ Liotyphlops anops

Taxon classificationAnimaliaSquamataAnomalepididae

(Cope, 1899)

24CF3A26-0C61-5E3C-861D-8D27B6FE9014

**Figure 7.**
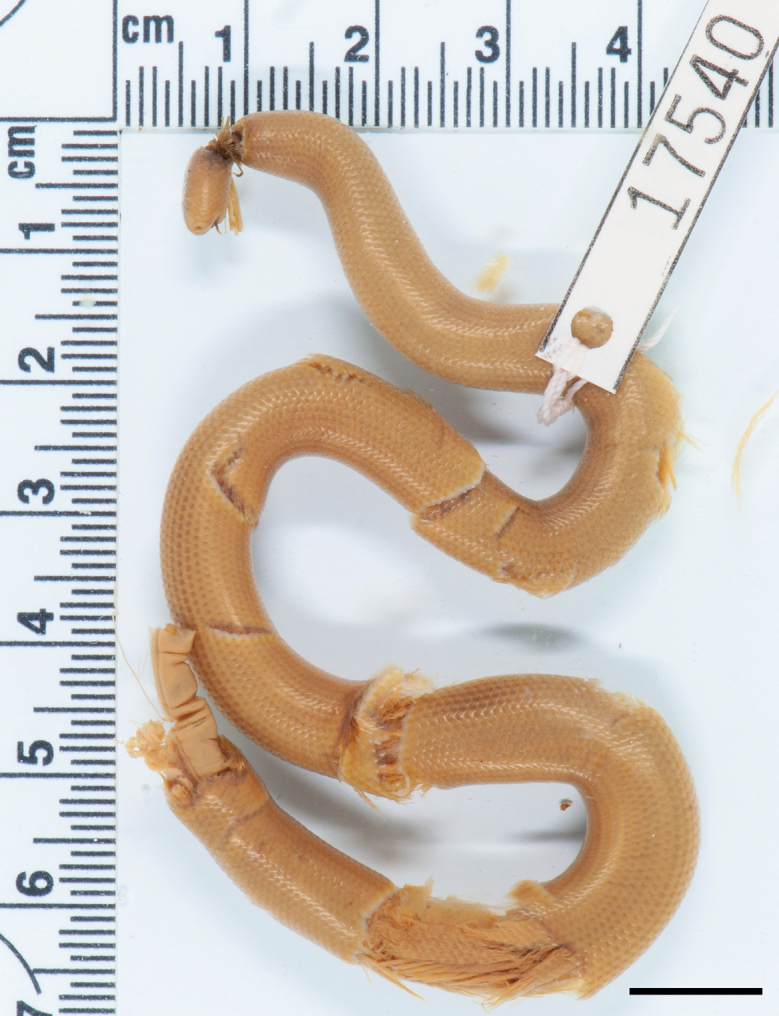
Lectotype of *Liotyphlopsanops*, AMNH R-17540, approximately 200 mm TL, Colombia, neighborhood of Bogota. Scale bar: 10 mm.

**Figure 8.**
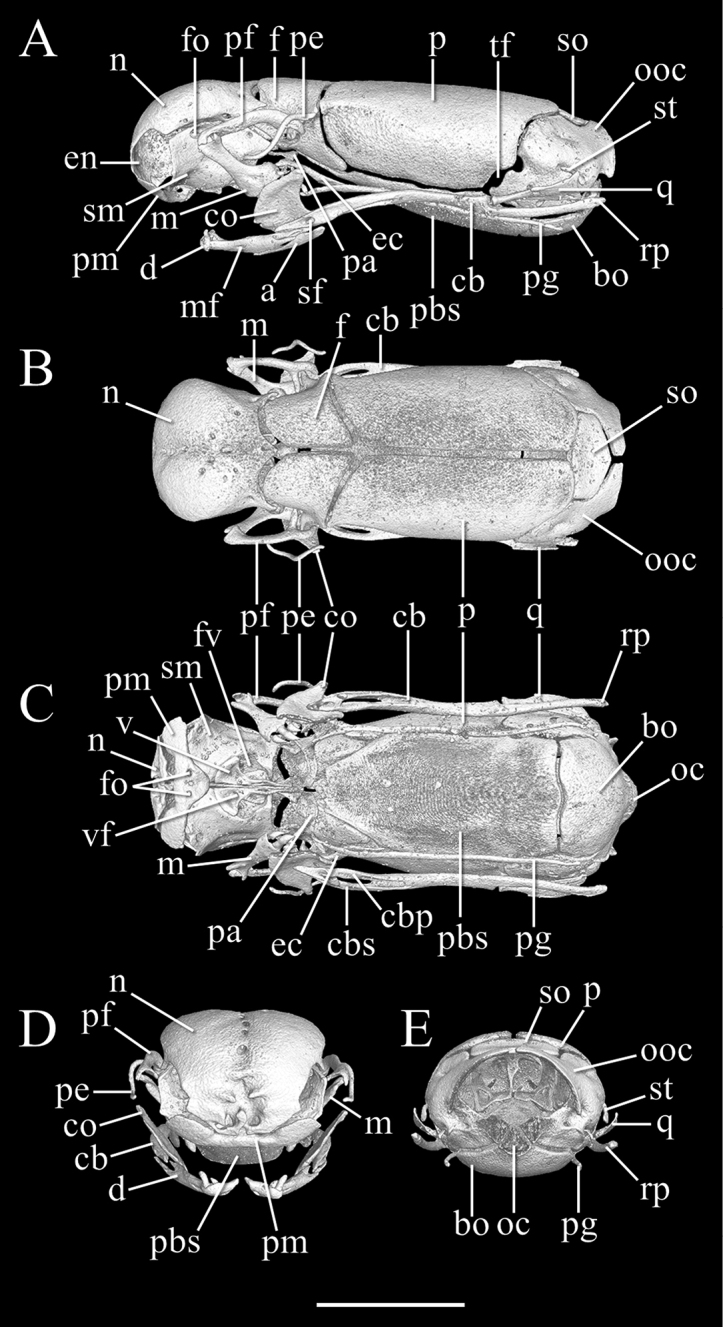
Three-dimensional reconstruction of the skull of *Liotyphlopsanops*, MCZ R-67936, based on HRXCT data. **A** lateral view **B** dorsal view **C** ventral view with lower jaw partially digitally removed **D** anterior view **E** posterior view. Scale bar: 1 mm. Anatomical abbreviations: **a**: angular; **bo**: basioccipital; **cb**: compound bone; **co**: coronoid; **d**: dentary; **ec**: ectopterygoid; **en**: external naris; **f**: frontal; **fo**: foramen; **fv**: fenestra vomeronasalis; **m**: maxilla; **mf**: mental foramen; **n**: nasal; **oc**: occipital condyle; **ooc**: otico-occipital (fused prootic + opisthotic + exoccipital); **p**: parietal; **pa**: palatine; **pbs**: parabasisphenoid; **pe**: postorbital element; **pf**: prefrontal; **pg**: pterygoid; **pm**: premaxilla; **q**: quadrate; **rp**: retroarticular process; **sf**: surangular foramen; **sm**: septomaxilla; **st**: supratemporal; **so**: supraoccipital; **tf**: trigeminal foramen; **v**: vomer; **vf**: vomerine foramen.

**Figure 9.**
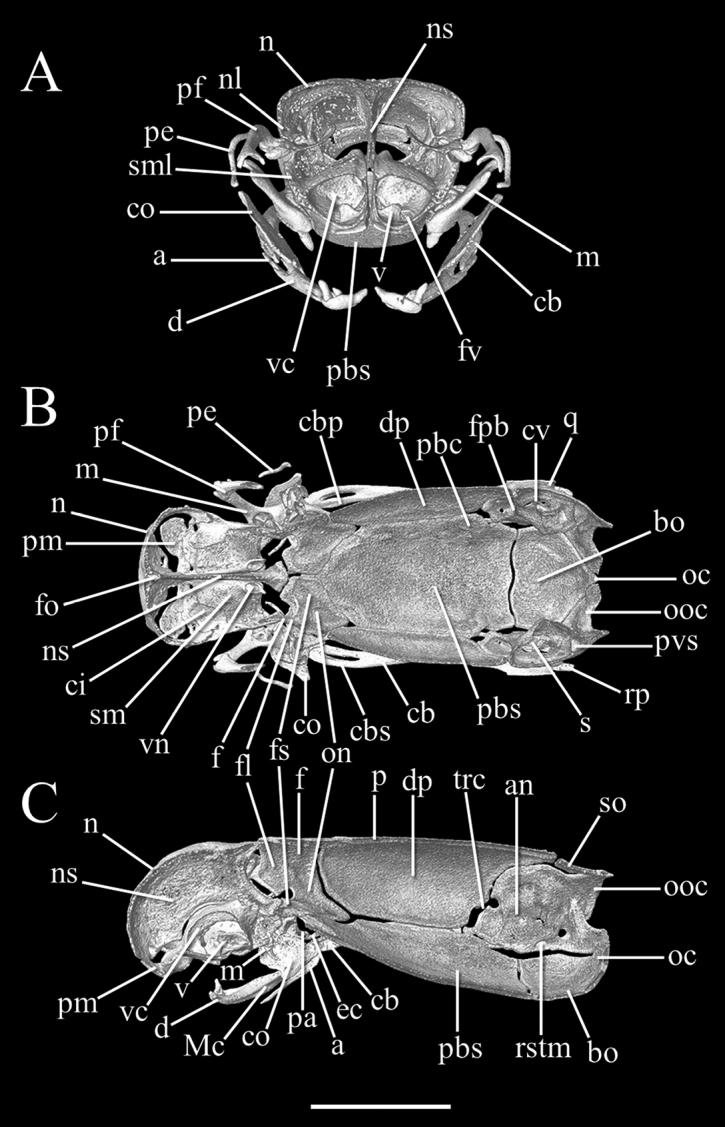
Three-dimensional reconstruction of the skull of *Liotyphlopsanops*, MCZ R-67936, based on HRXCT data. **A** transversal view **B** frontal view **C** sagittal view. Scale bar: 1 mm. Anatomical abbreviations: **a**: angular; **an**: acoustic nerve foramen; **bo**: basioccipital; **cb**: compound bone; **cbp**: compound bone prearticular component; **cbs**: compound bone surangular component; **ci**: conchal invagination; **co**: coronoid; **cv**: cavum vestibuli; **d**: dentary; **dp**: descensus parietalis; **ec**: ectopterygoid; **en**: external naris; **f**: frontal; **ﬂ**: frontal laterally descending ﬂange; **fo**: foramen; **fpb**: facial nerve palatine branch foramen; **fs**: frontal subolfactory process; **fv**: fenestra vomeronasalis; **m**: maxilla; **Mc**: Meckel’s canal; **mf**: mental foramen; **n**: nasal; **nl**: nasal lateral ﬂange; **ns**: medial nasal septum; **oc**: occipital condyle; **on**: optic nerve foramen; **ooc**: otico-occipital (fused prootic + opisthotic + exoccipital); **p**: parietal; **pa**: palatine; **pbc**: parabasal (Vidian) canal; **pbs**: parabasisphenoid; **pe**: postorbital element; **pf**: prefrontal; **pg**: pterygoid; **pm**: premaxilla; **pvs**: posterior vertical semicircular canal; **q**: quadrate; **rp**: retroarticular process; **rstm**: recessus scalae tympani medial aperture; **s**: stapes; **sf**: surangular foramen; **sm**: septomaxilla; **sml**: septomaxilla lateral ﬂange; **st**: supratemporal; **so**: supraoccipital; **tf**: trigeminal foramen; **trc**: trigeminofacialis chamber; **v**: vomer; **vc**: vomeronasal cupola; **vf**: vomerine foramen; **vn**: vomeronasal nerve passage.

**Figure 10.**
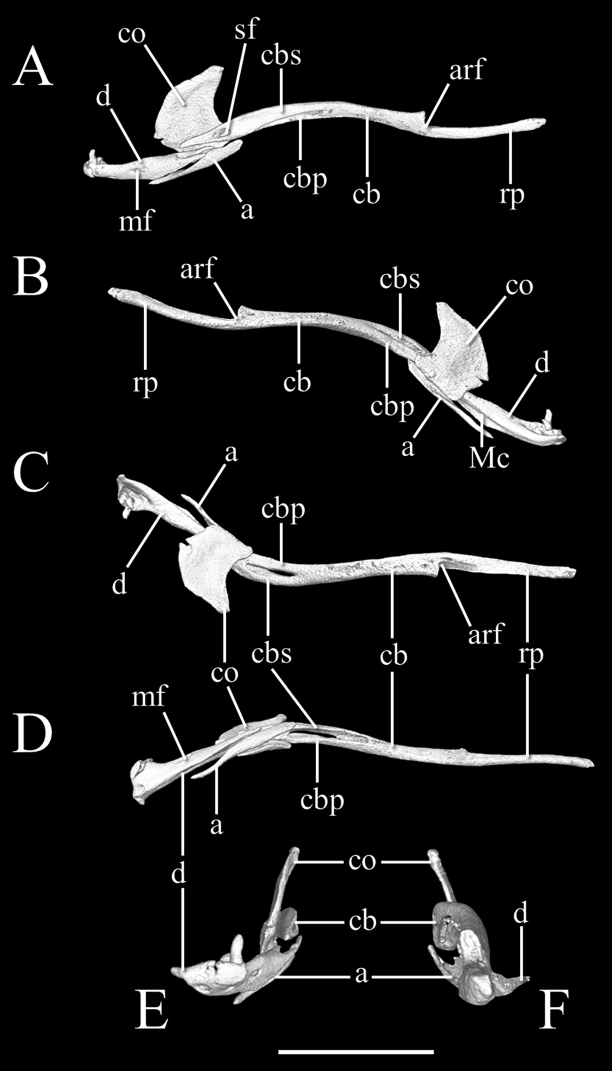
Three-dimensional reconstruction of the lower jaw of *Liotyphlopsanops*, MCZ R-67936, based on HRXCT data. **A** lateral view **B** medial view **C** dorsal view **D** ventral view **E** anterior view **F** posterior view. Scale bar: 1 mm. Anatomical abbreviations: **a**: angular; **arf**: articular fossa; **cb**: compound bone; **cbp**: compound bone prearticular component; **cbs**: compound bone surangular component; **co**: coronoid; **d**: dentary; **Mc**: Meckel’s canal; **mf**: mental foramen; **rp**: retroarticular process; **sf**: surangular foramen.

Figs 6 , 7 , 8 , 9 , 10 , Table 1

Helminthophis anops Cope, 1899: 10, pl. 4 fig. la–f. Type locality: “New Grenada”, Colombia. According to McDiarmid et al. (1999), Dunn (1944: 48) listed the type locality as “near Bogota”. The latter was the specific locality mentioned on the first page of Cope’s (1899: 3) posthumous publication and the source of much of the material.
Liotyphlops anops Dunn 1944: 48.
Liotyphlops metae –Dunn 1944: 49, figs 3, 4. Holotype: MLS 8. Type locality: “Villavicencio, Meta [Colombia], 498 meters”. Placed in synonymy by Dixon and Kofron (1984: 259).

##### Type material.

***Lectotype*.** AMNH R-17540, at least 200 mm TL (estimated from Fig. 7; specimen broken); type locality: Colombia, neighborhood of Bogota. **Lectotype by present designation**.

##### Diagnosis.

*Liotyphlopsanops* is distinguished from *L.albirostris*, *L.beui*, *L.bondensis*, *L.caissara*, *L.haadi*, *L.schubarti*, *L.taylori*, *L.ternetzii*, and *L.wilderi* in having four (vs three) scales contacting the posterior edge of the prefrontal. It is further distinguished from *L.argaleus* and *L.trefauti* in having two scales (vs one scale) contacting the posterior edge of the nasal between the second supralabial and the prefrontal. It is distinguished from *L.palauophis* sp. nov. in having the frontal scale single and 26/24/24 scales around the body (vs frontal scale divided and 28/26/26 scales around the body, and from *L.sousai* in having 562–597 dorsal scales and 531–572 ventral scales (vs 439 dorsal scales and 427 ventral scales).

##### Redescription.

Meristic data in Table 1. Total length 186.2–337.7 mm, head length 3.2–4.4 mm (1.3–1.7% TL), snout–vent length 184–332 mm (98.3–98.8% TL), tail length 2.2–5.7 mm (1.2–1.7% TL), head width 2.5–3.7 mm (78.1–85.7% HL), and head height 1.8–2.8 mm (56.2–63.6% HL). Body covered with cycloid scales. Rostral large, longer than wide, contacting nasals anterolaterally, prefrontals laterally, and single frontal posteriorly. Pair of triangular prefrontals, bordered anterolaterally by rostral, ventrally by large divided nasal, and dorsoposteriorly by frontal. Posterior edge of prefrontals passing posterior edge of rostral. Divided nasal scale bordered anteriorly by rostral, dorsally by prefrontal, ventrally by first and second supralabials, and posteriorly by two scales that lie between prefrontal and second supralabial. Eye spot poorly visible. Four scales contacting posterior edge of prefrontal (three cycloid scales + frontal). Two scales contacting posterior edge of nasal between second supralabial and prefrontal. Five or six scales in first vertical row of dorsal scales. Mental triangular, not divided, wider than long, contacting first infralabials. Supralabials four, infralabials three. Scales around body 26/24/24. Dorsal scales 562–597, ventral scales 531–572, and subcaudal scales 12–14.

##### Coloration in alcohol.

Dorsal and ventral body brown to pale cream. Head pale cream. Scales near opening of cloaca pale cream.

##### Description of skull.

High-resolution x-ray computed tomography of skull bones in Figs 8–10. Main body of premaxilla on ventral surface of snout. Maxilla–premaxilla contact widely separated. Lateral maxillary foramina absent. Maxilla alveolar row oriented transversely. Nasal fused. Nasal–frontal boundary convex posteriorly in shallow W-shaped suture. Prefrontal separated from nasal. Prefrontal moveably articulated to frontal. Postorbital element present. Posterior orbital margin incomplete. Frontals gradually tapering anteriorly. Frontal paired. Frontal–parietal contact (dorsal aspect) anteriorly concave, frontals extending posteriorly into broad median embayment in parietals. Parietal paired. Posterior border of parietal in contact with otico–occipital. Supraoccipital present and fused not participating in internal sidewall of neurocranium. Supratemporal present. Posteromedial flange of septomaxilla short, not contacting frontal. Septomaxilla with lateral flange contributing to posterior border of external naris. Fenestra for duct of Jacobson’s organ posteroventrally positioned. Palatine not in contact with vomer, maxilla, or pterygoid. Ectopterygoid present. Splenial not present as discrete element. Coronoid and angular separated by prearticular portion of compound bone. Retroarticular process long, longer than articular facet. Teeth present in maxilla and dentary, but lacking in premaxilla, palatine, and pterygoid.

##### Distribution.

Central Colombia (neighborhood of Bogota and Villavicencio in the department of Meta) (Fig. 6).

#### ﻿ Liotyphlops ternetzii

Taxon classificationAnimaliaSquamataAnomalepididae

(Boulenger, 1896)

AD0FFE16-85BC-538A-8EB0-7CE4E2858AF6

**Figure 11.**
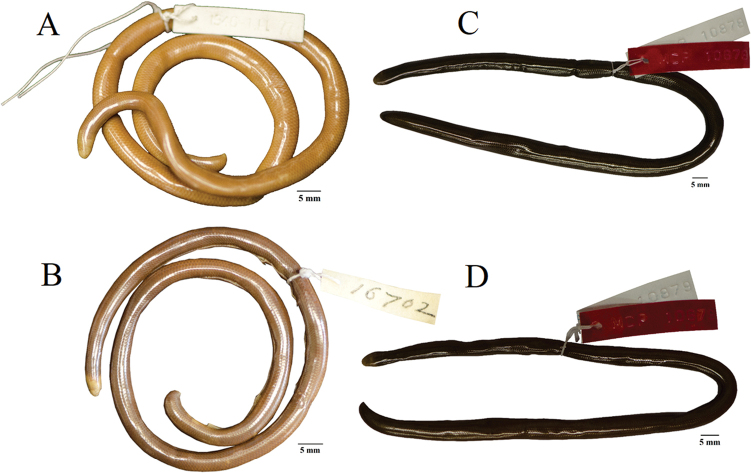
Types of *Liotyphlopsternetzii* and *Liotyphlopsbeui*. **A** holotype of *L.ternetzii* (BMNH 1946.1.11.77, 325.1 mm TL) from Paraguay **B** paratype of *L.beui* (MCZ 16702, 279.2 mm TL) from Butantan, São Paulo, Brazil **C** specimen of *L.ternetzii* (MCP 10878, 248.9 mm TL) with dark brown coloration **D** specimen of *L.beui* (MCP 10879, 233.9 mm TL) with dark brown coloration.

**Figure 12.**
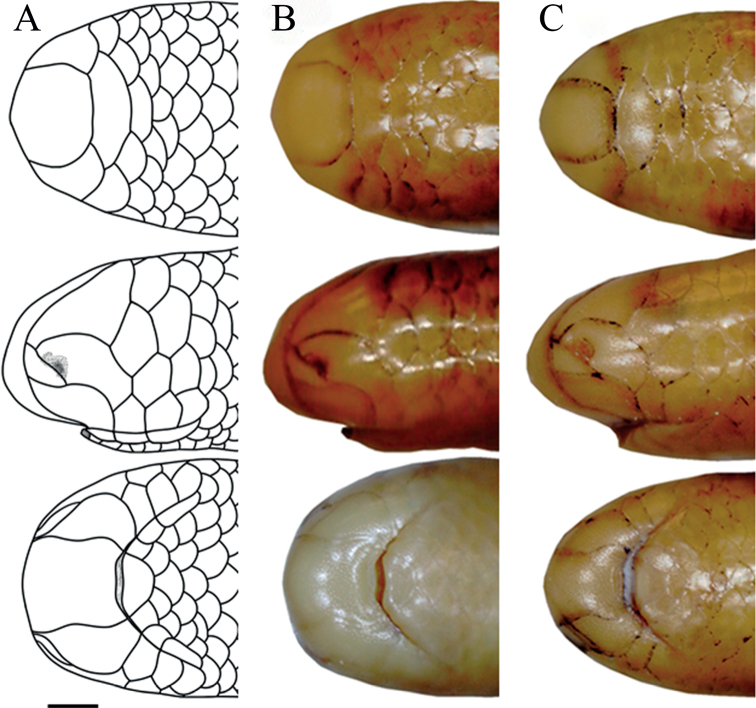
Dorsal, lateral, and ventral views of the head of *Liotyphlops*. **A***L.ternetzii*, holotype (BMNH 1946.1.11.77), drawing **B***L.ternetzii*, holotype **C***L.beui* (MCZ 16702) paratype. Scale bar: 1 mm.

**Figure 13.**
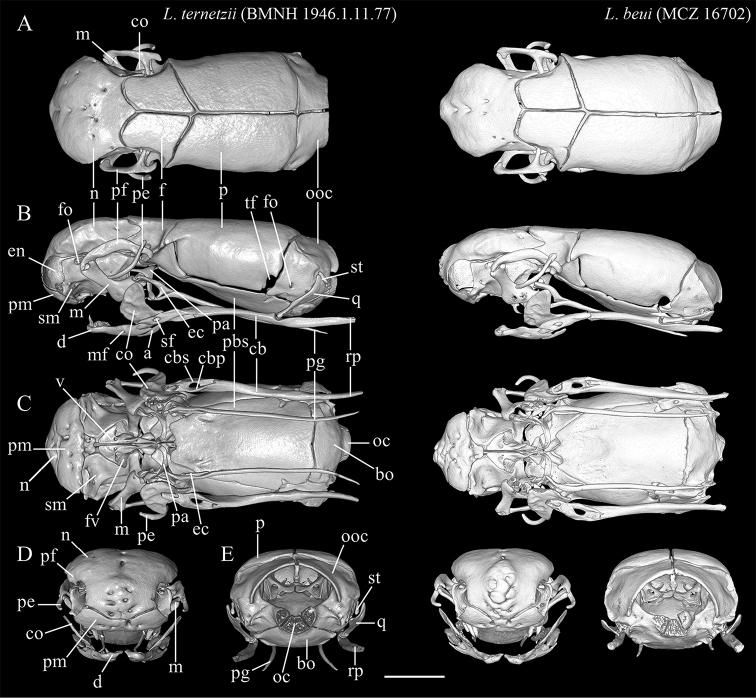
Three-dimensional reconstruction of the skull of holotype *Liotyphlopsternetzii* (BMNH 1946.1.11.77), and of the skull of the paratype of *Liotyphlopsbeui* (MCZ 16702) based on HRXCT data. **A** dorsal view **B** lateral view **C** ventral view **D** anterior view **E** posterior view. Scale bar: 1 mm. Anatomical abbreviations: **a**: angular; **bo**: basioccipital; **cb**: compound bone; **cbp**: compound bone prearticular component; **cbs**: compound bone surangular component; **co**: coronoid; **d**: dentary; **ec**: ectopterygoid; **en**: external naris; **f**: frontal; **fo**: foramen; **m**: maxilla; **mf**: mental foramen; **n**: nasal; **oc**: occipital condyle; **ooc**: otico-occipital (fused prootic + opisthotic + exoccipital); **p**: parietal; **pa**: palatine; **pbs**: parabasisphenoid; **pe**: postorbital element; **pf**: prefrontal; **pg**: pterygoid; **pm**: premaxilla; **q**: quadrate; **rp**: retroarticular process; **sm**: septomaxilla; **sf**: surangular foramen; **st**: supratemporal; **tf**: trigeminal foramen; **v**: vomer.

**Figure 14.**
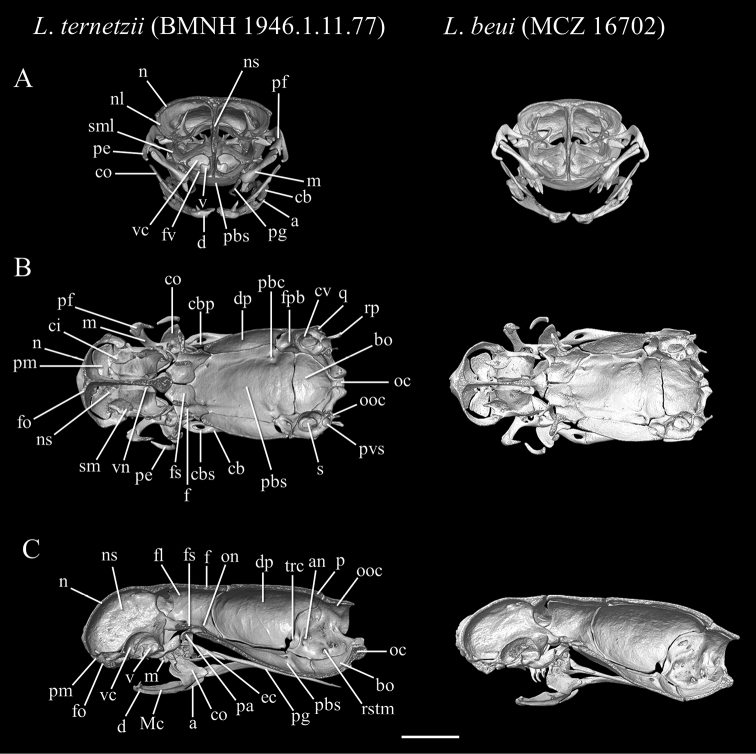
Three-dimensional reconstruction of the skull of holotype *Liotyphlopsternetzii* (BMNH 1946.1.11.77), and of the skull of the paratype of *Liotyphlopsbeui* (MCZ 16702) based on HRXCT data. **A** transversal view **B** frontal view **C** sagittal view. Scale bar: 1 mm. Anatomical abbreviations: **a**: angular; **an**: acoustic nerve foramen; **bo**: basioccipital; **cb**: compound bone; **cbp**: compound bone prearticular component; **cbs**: compound bone surangular component; **ci**: conchal invagination; **co**: coronoid; **cv**: cavum vestibuli; **d**: dentary; **dp**: descensus parietalis; **ec**: ectopterygoid; **en**: external naris; **f**: frontal; **ﬂ**: frontal laterally descending ﬂange; **fo**: foramen; **fpb**: facial nerve palatine branch foramen; **fs**: frontal subolfactory process; **fv**: fenestra vomeronasalis; **m**: maxilla; **Mc**: Meckel’s canal; **mf**: mental foramen; **n**: nasal; **nl**: nasal lateral ﬂange; **ns**: medial nasal septum; **oc**: occipital condyle; **on**: optic nerve foramen; **ooc**: otico-occipital (fused prootic + opisthotic + exoccipital); **p**: parietal; **pa**: palatine; **pbc**: parabasal (Vidian) canal; **pbs**: parabasisphenoid; **pe**: postorbital element; **pf**: prefrontal; **pg**: pterygoid; **pm**: premaxilla; **pvs**: posterior vertical semicircular canal; **q**: quadrate; **rp**: retroarticular process; **rstm**: recessus scalae tympani medial aperture; **s**: stapes; **sf**: surangular foramen; **sm**: septomaxilla; **sml**: septomaxilla lateral ﬂange; **st**: supratemporal; **so**: supraoccipital; **tf**: trigeminal foramen; **trc**: trigeminofacialis chamber; **v**: vomer; **vc**: vomeronasal cupola; **vf**: vomerine foramen; **vn**: vomeronasal nerve passage.

**Figure 15.**
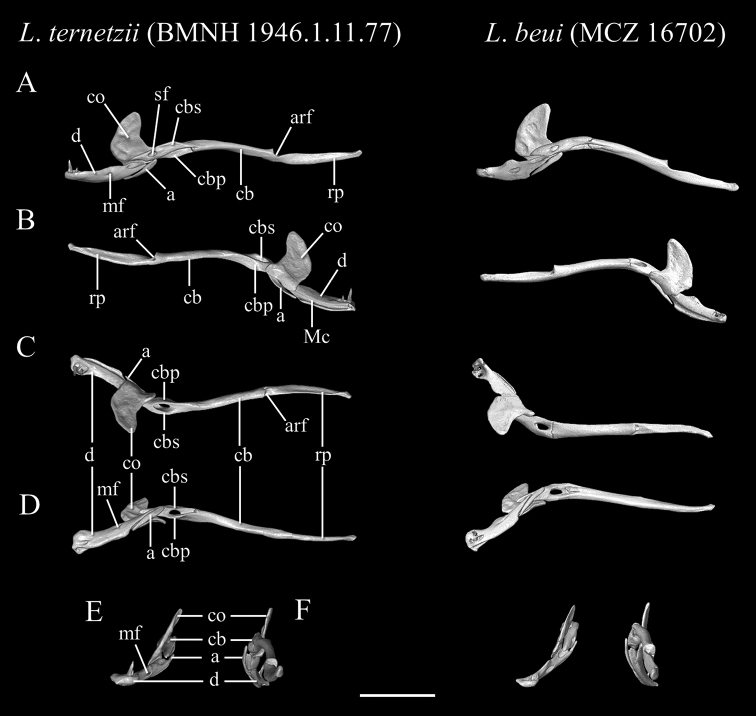
Three-dimensional reconstruction of the lower jaw of *Liotyphlopsternetzii*, BMNH 1946.1.11.77, holotype, and *Liotyphlopsbeui*, MCZ 16702, paratype, based on HRXCT data. **A** lateral view **B** medial view **C** dorsal view **D** ventral view **E** anterior view **F** posterior view. Scale bar: 1 mm. Anatomical abbreviations: **a**: angular; **arf**: articular fossa; **cb**: compound bone; **cbp**: compound bone prearticular component; **cbs**: compound bone surangular component; **co**: coronoid; **d**: dentary; **Mc**: Meckel’s canal; **mf**: mental foramen; **rp**: retroarticular process; **sf**: surangular foramen.

**Figure 16.**
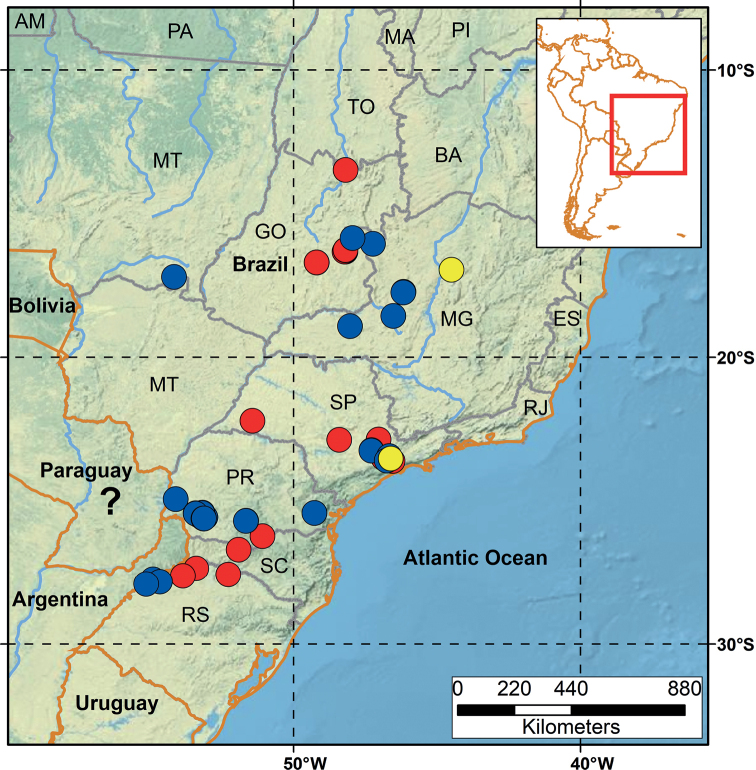
Localities of specimens originally identified as *Liotyphlopsternetzii* (blue dots) and *Liotyphlopsbeui* (red dots) examined in this study. Paratypes of *L.beui* (yellow dots), and holotype of *L.ternetzii* (? = undetermined type locality).

Figs 11 , 12 , 13 , 14 , 15 , 16 , Tables 1 , 2

Helminthophis ternetzii Boulenger, 1896: 584. Holotype: BMNH 1946.1.11.77. Type locality: Paraguay.
Helminthophis incertus Amaral, 1924: 29. Holotype: MCZ R17846. Type locality: Surinam [Suriname]. Placed in synonymy by Dixon and Kofron (1984 [dated 1983]: 255–256), who also rejected the type locality as Suriname.
Helminthophis beui Amaral, 1924: 25–30. Holotype: IB 1806. Type locality: Butantan, São Paulo, Brazil. syn. nov.
Helminthophis collenettei Parker, 1928: 97. Holotype: BMNH 1946.1.10.73 (formerly BMNH 1928.1.12.1). Type locality: Burity, 2250 ft., 30 miles northeast of Coyaba [Cuiabá], Mato Grosso [Brazil]. Placed in synonymy by Amaral (1954: 192).
[Liotyphlops] incertus–Vanzolini 1948: 380.
[Liotyphlops] ternetzi–Smith and Grant 1958: 207.
Liotyphlops ternetzii –Peters and Orejas-Miranda 1970: 183, in part; included L.beui in the synonymy.
Liotyphlops ternetzii –McDiarmid et al. 1999: 51–52.
Liotyphlops ternetzii –Wallach et al. 2014: 397–398.

##### Type material.

***Holotype*.** BMNH 1946.1.11.77, 325.1 mm TL; type locality: Paraguay.

##### Diagnosis.

*Liotyphlopsternetzii* is distinguished from *L.anops*, *L.argaleus*, *L.sousai*, and *L.trefauti* in having three (vs four) scales contacting the posterior edge of the prefrontal scale. It is distinguished from *L.albirostris*, *L.bondensis*, *L.caissara*, *L.haadi*, and *L.wilderi* in having two scales (vs one scale) contacting the posterior edge of the nasal between the second supralabial and the prefrontal. It is distinguished from *L.taylori* by having three (vs two) infralabial scales, and from *L.palauophis* sp. nov. in having a single frontal scale (vs frontal scale divided). Is distinguished from *L.schubarti* in the pale cream, dark brown, or black coloration (vs light brown).

##### Redescription.

Meristic data in Tables 1, 2. Total length of holotype 325.1 mm, head length 4.8 mm (1.5% TL), snout–vent length 317 mm (97.5% TL), tail length 8.1 mm (2.5% TL), head width 3.6 mm (75% HL), and head height 2.7 mm (56.3% HL). Body covered with cycloid scales. Snout rounded, rostral scale large, longer than wide, contacting nasals anterolaterally, prefrontals laterally, and single frontal posteriorly. Pair of triangular prefrontals, bordered anterolaterally by rostral, ventrally by large divided nasal, and dorsoposteriorly by frontal. Posterior edge of prefrontals passing posterior edge of rostral. Nasal scale divided and bordered anteriorly by rostral, dorsally by prefrontal, ventrally by first and second supralabials, and posteriorly by two scales located between prefrontal and second supralabial. Eye spot poorly visible. Three scales contacting posterior edge of prefrontal (two cycloid scales + frontal). Two scales contacting posterior edge of nasal between second supralabial and prefrontal. Five scales in first vertical row of dorsal scales. Mental triangular, not divided, wider than long, contacting first infralabial. Supralabial scales four, infralabial scales three. Scales around body 24/22/21. Dorsal scales 475, ventral scales 452, and subcaudal scales 20.

**Table 2.** Meristic characters of specimens identified as *Liotyphlopsbeui* and *L.ternetzii*, presented as ranges with minimum, maximum, and mode in parentheses. **SPEP** = number of scales contacting posterior edge of prefrontal; **SPEN** = number of scales contacting posterior edge of nasal between second supralabial and prefrontal; **SFVRD** = number of scales in the first vertical row of dorsals; **SL** = number of supralabial scales; **IL** = number of infralabial scales; **ASR** = number of anterior scale rows around body; **MSR** = number of scale rows around the midbody; **PSR** = number of posterior scale rows around body; **DSR** = number of dorsal scale rows; **VSR** = number of ventral scales rows; **SC** = number of subcaudal scales; **n** = number of specimens examined in this study; **(p)** = *L.beui* paratypes; **(h)** = *L.ternetzii* holotype.

Species/Count	n	SPEP	SPEN	SFVRD	SL	IL	ASR	MSR	PSR	DSR	VSR	SC
*L.beui*	50	3–3(3)	2–2(2)	5–6(5)	4–4(4)	3–3(3)	22–26(22)	20–22(22)	20–22(20)	366–532(453)	348–511(364)	11–22(12)
*L.beui* **(p)**	2	3–3(3)	2–2(2)	5–5(5)	4–4(4)	3–3(3)	22–22(22)	20–20(20)	20–20(20)	462–477	439–452	19–20
*L.ternetzii*	50	3–3(3)	2–2(2)	5–6(5)	4–4(4)	3–3(3)	22–26(22)	20–23(20)	20–22(20)	353–539(417)	341–514(381)	11–22(15)
*L.ternetzii* **(h)**	1	3	2	5	4	3	24	22	21	475	452	20

##### Coloration in alcohol.

Dorsal and ventral body pale cream. Scales near opening of cloaca and subcaudal scales lighter than rest of body.

##### Description of skull.

High-resolution x-ray computed tomography of skull bones in Figs 13–15. Main body of premaxilla on ventral surface of snout. Maxilla–premaxilla contact widely separated. Lateral maxillary foramina absent. Maxilla alveolar row oriented transversely. Nasal fused. Nasal–frontal boundary convex posteriorly in shallow W-shaped suture. Prefrontal separated from nasal. Prefrontal moveably articulated to frontal. Postorbital element present. Posterior orbital margin incomplete. Frontals gradually tapering anteriorly. Frontal paired. Frontal–parietal contact (dorsal aspect) anteriorly concave, i.e., frontals extending posteriorly into broad median embayment in parietals. Parietal paired. Posterior border of parietal in contact with otico–occipital. Supraoccipital absent. Supratemporal present. Posteromedial flange of septomaxilla short, not contacting frontal. Septomaxilla with lateral flange contributing to posterior border of external naris. Fenestra for duct of Jacobson’s organ posteroventrally positioned. Palatine not in contact with vomer, maxilla, or pterygoid. Ectopterygoid present. Splenial not present as discrete element. Coronoid and angular separated by prearticular portion of compound bone. Retroarticular process long, longer than articular facet. Teeth present in maxilla and dentary, but lacking in premaxilla, palatine, and pterygoid.

##### Distribution.

Known from Brazil (Mato Grosso, Goiás, Minas Gerais, São Paulo, Paraná, Santa Catarina, and Rio Grande do Sul), Paraguay (Amambay, Caazapá, Canendiyu, Itapúa, Presidente Hayes), Uruguay (Río Negro, Salto), and Argentina (Corrientes, Entre Ríos, Formosa, Jujuy, Misiones, Salta) (Fig. 16). In the original description, the locality of the holotype is described as Paraguay.

## ﻿Discussion

The description of new species based on single specimens is generally discouraged due to the obvious limitations, for example, in describing variation and geographical distribution (Santos and Reis 2018). More material will provide data on morphological variation, as well as ecological information that may be useful in conservation efforts. The redescription here of the lectotype of *L.anops* (AMNH R-17540) was based on photographs sent by the curators of the AMNH due to the great fragility of the specimen, making impossible the packing, transport, and the use of invasive techniques. The examination of these photographs of the lectotype was complemented by data obtained by the examination of other specimens of *L.anops*, providing the redescription with data of external morphology and osteology of the skull.

The specimens of *L.beui* (two paratypes and 50 non-types) and *L.ternetzii* (the holotype and 50 non-types) examined (Figs 11–16) showed limited variation in meristic characters (Table 2), which does not warrant the recognition of these taxa as separate species. The number and disposition of head scales do not distinguish the two taxa (Fig. 12): (1) three scales contacting the posterior edge of the prefrontal; (2) two scales contacting the posterior edge of the nasal between the second supralabial and the prefrontal; (3) five or six scales in the first vertical row of dorsal scales; (4) four supralabial scales, and (5) three infralabial scales. The supratemporal bone of anomalepidid snakes is either very reduced or absent (*Anomalepis*), and the high-resolution x-ray tomography showed that the two paratypes of *L.beui* (MCZ R-16702 and MCZ R-17842) lack a supratemporal, which is instead present, although highly reduced, in all other specimens of *L.beui* scanned and examined.

*Liotyphlopsbeui* was removed from the synonymy of *L.ternetzii* by Dixon and Kofron (1984) based on two characters: (1) 20 scale rows posteriorly around the body (22 in *L.ternetzii*), and (2) a dorsal scale count of 384–455 (463–510 in *L.ternetzii*). In my sample, however, *L.beui* had 366–532 (mode 453), while *L.ternetzii* had 353–539 (mode 417), with the two ranges completely overlapping; and dorsal scale count in *L.beui* 20–22 (mean 22) and in *L.ternetzii* 20–23 (mean 20), but the holotype of *L.ternetzii* has 22 (Table 2). In addition, all other meristic characters (Table 2), coloration pattern, and an extensive study of skull bone characters showed no significant variation that can be used as diagnostic characters for *L.beui*. After a detailed morphological examination of specimens of *L.beui* and *L.ternetzii*, including the relevant type materials, *L.beui* is considered a junior synonym of *L.ternetzii*.

It is important to highlight that, in view of the limitation of diagnostic phenotypic characters for species of the genus *Liotyphlops* and the lack of knowledge about the evolutionary relationships of their species, there is a need for fieldwork to collect samples of fresh tissue to obtain genetic material, which will allow studying the systematics and testing the limits of *Liotyphlops* species from a molecular perspective.

## Supplementary Material

XML Treatment for Liotyphlops palauophis

XML Treatment for Liotyphlops anops

XML Treatment for Liotyphlops ternetzii

## ﻿Appendix I

**Examined specimens**

***Liotyphlopsalbirostris***: Colombia. Bolívar, Arjona: CM 39565. Panama. Herrera, Santa María: CM 44652. Venezuela. Distrito Capital, road below La Guaira, km 5, East of Caracas: CM 90256. Distrito Capital, Libertador: MHNLS 514. Miranda, Urbanización Altamira: MHNLS 11824. Urbanización Macaracuay: MHNLS 15550.

***Liotyphlopsanops***: Colombia. Neighborhood of Bogota: lectotype AMNH R-17540. Meta, Villavicencio: MCZ R-67936, MCZ R-67937, MZUSP-S 5998.

***Liotyphlopsargaleus***: Colombia. Meta, La Selva: MCZ R-66383 paratype.

***Liotyphlopsbeui***: Brazil. Goiás, Goiânia: CEPB 1398, CEPB 1422, CEPB 2491, CEPB 3610. Luziânia: CEPB 6601, CEPB 6602, CEPB 6603, CEPB 6604, CEPB 6900, CEPB 6901, CEPB 6902, CEPB 6903, CEPB 6904, CEPB 6905, CEPB 6642, CEPB 6643, CEPB 6646, CEPB 6651, CEPB 6659, CEPB 6672, CEPB 8849. Minaçu: CEPB 8409. São Paulo, Botucatu: MNRJ 23247. Campinas: MNRJ 8143. Carapicuíba: MCP 16361, MNRJ 10578. Itu: MNRJ 8144. Pirapozinho: MNRJ 22022. São Caetano do Sul: MCP 16365. São Paulo: MCP 16366, MCP 16368, MNRJ 10577, ZUFSM 1569. Instituto Butantan, paratypes MCZ R-16702, MCZ R-17842. Paraná, Boa Vista da Aparecida: MCP 10853, MCP 10855, MCP 10854, MCP 10879. Cruzeiro do Iguaçu: MCP 10880. Curitiba: MCP 16362, MCP 16363. Três Barras do Paraná: MCP 10857, MCP 10858, MCP 10859, MCP 10862, MCP 10864. União da Vitótia: MCP 16360. Santa Catarina, Passos Maia: UFRGS 6275. Rio Grande do Sul, Erechim: UFRGS 6494. Frederico Westphalen: MCP 9494. Bom Progresso: MCP 19086.

***Liotyphlopshaadi***: Colombia. Amazonas Department, middle region of the Caquetá River, La Pedrera district: IAvH 5434 holotype. Leticia, Vereda de los Lagos: IAvH 5435 paratype.

***Liotyphlopspalauophis* sp. nov.**: Colombia. Neighborhood of Bogota: holotype AMNH R-09550.

***Liotyphlopsschubarti***: Brazil. São Paulo, Campinas: ZUEC REP 2278, ZUEC REP 2279, ZUEC REP 2280, ZUEC REP 2281. Sapucaí: MZUSP-S 4099.

***Liotyphlopssousai***: Brazil. Santa Catarina, Passos Maia: holotype, UFRGS 6274

***Liotyphlopstaylori***: Brazil. Mato Grosso, Porto Estrela: holotype, MZUSP-S 14975

***Liotyphlopsternetzii***: Paraguay. holotype, BMNH 1946.1.11.77. Brazil. Mato Grosso, Itiquira: UFRGS 6458. Distrito Federal, Brasília: MCP 18381. Minas Gerais, Cabeceira Grande: MCP 19228. Indianópolis: MNRJ 8147. João Pinheiro: MNRJ 11329, MNRJ 14957. Patos de Minas: MNRJ 17300. São Paulo, Itu: MCP 10699. São Paulo: MCP 3680, MCP 6986. Taboão da Serra: MCP 7349. Paraná, Boa Vista da Aparecida: MCP 10849, MCP 10869, MCP 10870, MCP 10878, MCP 10850, MCP 10851, MCP 10852. Curitiba: MCP 1943. Cruzeiro do Iguaçu: MCP 10847, MCP 10872, MCP 10873, MCP 10874, MCP 10875, MCP 10876, MCP 10877, MCP 10881, MCP 10882, MCP 10883, MCP 10885, MCP 10886. Diamante D’Oeste: MCP 16364. Pinhão: MCP 7186, MCP 7195, MCP 7196, MCP 7197, MCP 7198, MCP 7199, MCP 7361. Três Barras do Paraná: MCP 10856, MCP 10860, MCP 10861, MCP 10863, MCP 10865, MCP 10866, MCP 10867, MCP 10884. Rio Grande do Sul, Porto Vera Cruz: MCP 11676. Porto Xavier: MCP 11706. Santo Cristo: MCP 11661.

***Liotyphlopswilderi***: Brazil. Bahia, Itapebi: MNRJ 15657. Minas Gerais, Caeté: MNRJ 20633, MZUSP-S 3842.
